# Environmentally friendly catalyst- and solvent-free synthesis of 2-anilino nicotinic acids derivatives as potential lead COX inhibitors

**DOI:** 10.1186/s13065-023-01078-y

**Published:** 2023-11-20

**Authors:** Mahsa Yarhorhosseini, Shahrzad Javanshir, Ahmad Shahir Sadr, Milad Noori, Navid Dastyafteh, Maryam Esmkhani, Aida Iraji, Mohammad Mahdavi

**Affiliations:** 1 Heterocyclic Chemistry Research Laboratory, Department of Chemistry, Iran University of Science and Technology, Tehran, 16846-13114 Iran; 2 Bioinformatics Research Center, Cheragh Medical Institute & Hospital, Kabul, Afghanistan; 3 Stem Cells Technology Research Center, Shiraz University of Medical Sciences, Shiraz, Iran; 4 Central Research Laboratory, Shiraz University of Medical Sciences, Shiraz, Iran; 5 Endocrinology and Metabolism Research Center, Endocrinology and Metabolism Clinical Sciences Institute, Tehran University of Medical Sciences, Tehran, Iran

**Keywords:** 2-Anilino nicotinic acids, COX, Molecular dynamic simulations, Solvent -free synthesis, Similarity search

## Abstract

In this study, an environmentally friendly, solvent- and catalyst-free synthesis of 2-anilino nicotinic acids derivatives is reported. This operationally simple and green procedure was applied to a selection of primary aromatic amines giving rise to 23 derivatives of 2-anilino nicotinic acids in a very short reaction time (15–120 min) with good to excellent yield. Next, similarity searches were executed on these derivatives to find the possible biological target. These products were screened for inhibition of COX-1 and COX-2 by molecular docking and dynamic studies. In silico studies revealed that among these derivatives, the structure **10** bearing *meta*-chlorine substitutions could act as COX-1 and COX-2 inhibitors. These results can be used in designing important lead compounds for further development as potential anti-inflammatory drugs.

**Supplementary Information:**

The online version contains supplementary material available at 10.1186/s13065-023-01078-y.

## Introduction

**Fig. 1:**
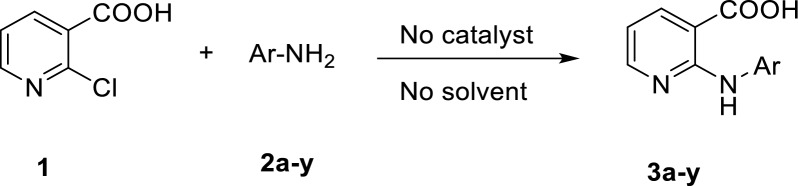
Amination of 2-chloronicotinic acid under catalyst-free solvent-free conditions

2-(arylamino)nicotinic acids known as a central core for the synthesis of a wide range of biologically active molecules with potential antibacterial [1], antiviral [2], antiallergic [3, 4], antitumor agents [5, 6] as well as nonsteroidal anti-inflammatory drugs, usually abbreviated as NSAIDs potencies [7, 8]. Generally, 2-arylaminonicotinic acids are synthesized by the Ullman reaction [9, 10]. Nonetheless, these processes require using a stoichiometric amount of copper reagent, a non-green solvent, and a long reaction time with generally average yields. In an alternative method, 2-(arylamino)nicotinic acids were synthesized by amination of 2-chloronicotinic acid in the presence of pyridine and *para*-toluenesulfonic acid under reflux conditions in water for very long reaction times [11]. Moreover, some of the methods were conducted in non-green solvents such as DMF [12, 13] or xylene [9] which are often harmful to the environment. Microwave irradiation has also been used with relatively expensive diisopropylethylamine as a base at 200 °C [14]. Consequently, given the problems that threaten our environment, it is necessary to find alternative methods for synthesizing 2-(arylamino)nicotinic acid and its derivatives to avoid all these drawbacks. Recently, solvent-free organic syntheses have received considerable attention because they are operationally simple, safer, and high-speed due to the high concentration of materials and more environmentally benign chemical processes. The simpler and cleanest work-up often involves nontoxic materials and produces excellent yields. Therefore, we report a facile, environmentally friendly, and practical procedure for synthesizing 2-(arylamino)nicotinic acids via amination of 2-chloronicotinic acid with appropriate amines under catalyst- and solvent-free conditions. Compared with conventional methods, the reaction yields under solvent-free conditions are greater, and the reaction time is shorter (Fig. 1).

Virtual screening (VS) advances during the past few years have established valuable tools in drug discovery in which an in silico method was executed to screen databases for bioactive molecules or biological targets. The VS can be applied using structure-based methods such as molecular docking or molecular dynamics simulations, and ligand-based techniques include similarity search and pharmacophore search [15].

NSAIDs are a class of drugs with analgesic (pain-killing) and antipyretic (fever-reducing) properties and, in higher doses, anti-inflammatory effects. NSAIDs inhibit the activity of cyclooxygenase (COX) enzymes and the synthesis of thromboxanes and prostaglandins (PGs), which are crucial mediators of inflammation [16, 17]. PGs were generated via multi-step process so that the phospholipid is metabolized to arachidonic acid by the catalytic activity of phospholipase A2 (PLA2) which is subsequently hydrolysis to prostaglandin H2 by the actions of both COX-1 and COX-2. PGs and thromboxane are eventually produced through the catalytic activities of PGs and TXA2 synthases in the downstream mechanism [18]. COX enzymes exist in two major isoforms, COX-1 and COX-2, in which their expression and roles in the body are mostly different. COX-1 is expressed in most tissues, predominantly in gastrointestinal (GI) mucosa, platelets, endothelium, kidneys, and the uterus. In GI, COX-1 synthesizes PGE2 and PGI2, which exert cytoprotective effects. COX-2 is always regarded as a pathologic enzyme chiefly responsible for inflammation. Also, it is thought that inhibiting COX-2 leads to anti-inflammatory and antipyretic effects; however, the concept that COX-2 is the only COX isoform involved in inflammation has also been challenged by several studies recently [19–22]. Lately, COX-1 has appeared as a noticeable player in CNS neuroinflammation [23–25]. It is believed that COX-1 is responsible for the primary prostanoid response to inflammatory stimuli, while COX-2 is a major factor in the synthesis of prostanoids expressly during inflammation [26–29].

**Fig. 2:**
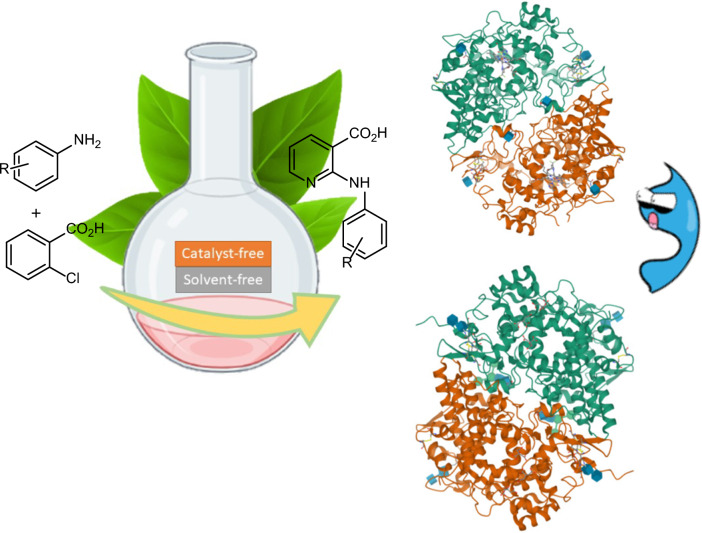
Catalyst and Solvent-Free Synthesis of 2-Anilino Nicotinic Acids Derivatives as Potential COX Inhibitors

As a result, in the current study, an environmentally friendly, practical procedure for synthesizing 2-(arylamino)nicotinic acids via amination of 2-chloronicotinic acid with appropriate amines under catalyst- and solvent-free conditions were developed. Next, in silico similarity searches were performed in different databases, and COX-enzymes were proposed as the possible biological target (Fig. 2). Next, molecular docking studies were executed to explore the potential binding affinities of synthesized compounds against the proposed biological target. Finally, molecular dynamics (MD) simulations were conducted against COX-enzymes to study the behavior of derivatives within the binding site of enzymes.

## Results and discussion

### Synthesis

In a first attempt, the reaction of 2-chloronicotinic acid (1) with aniline (2a) was examined under various reaction conditions for the optimization of reaction conditions, and the results were listed in Additional file 1: Table S1.

Initially, the effect of the catalyst was tested. Interestingly, the desired products were achieved in the absence of any catalyst (Additional file 1: Table S1, entries 1–5). Later, the effect of temperature (100–150 °C) and the solvent was examined, and the best yield is obtained at 120 °C solvent-free conditions (Additional file 1: Table S1, entries 6–13). Therefore, considering the viewpoints of green chemistry, the reaction of aniline derivatives 2a-m with 2-chloronicotinic acid (1) was carried out without using any catalyst under solvent-free conditions. Finally, the effect of the proportion of reactants was studied (Additional file 1: Table S1, entries 13–15). Consequently, the optimal conditions for obtaining (**3a**) were established, which involve heating the mixture at 120 °C under solvent-free conditions without using any catalyst when the ratio of 2- chloronicotinic acid to aniline (1/2a) is 1:1.5.

In the next stage, the scope of reaction for the synthesis of 2-arylaminonicotinic acids from various aromatic amines with electron-donating or electron-withdrawing was investigated. The results are listed in Additional file 1: Table S2. As shown in Additional file 1: Table S2, all of the reactions proceeded efficiently and the desired products were produced in good to excellent yields in relatively short reaction times, except in a few cases.

Electron-donating substituents on the aromatic ring of aromatic amines improve the nucleophilic aromatic substitution of 2-chloronicotinic acid and produce higher yields and electron-withdrawing substituents on the aromatic ring of aromatic amines decrease the yield. Note that there were no significant electronic effects in reactions of meta or *para*-substituted anilines, but the incorporation of an ortho electron-withdrawing substituent on the aniline was found to reduce the yield substantially. Moreover, it was found that the incorporation of both *ortho* substituents to aniline has impeded effective amination and reduced yields. However, the presence of one ortho substituent does not impede the effect amination of 2-chloronicotinic acid.

Additional file 1: Table S3 compares the efficiency of the present method with the efficiency of other methods in the synthesis of 2-(Arylamino) nicotinic acids. For this purpose, 3a was chosen as a model reaction and the comparison is in reaction times, reaction conditions and percentage yields.

The reaction mechanism is shown in Additional file 1: Fig. S1, where in the first step the reaction starts with nucleophilic addition of aryl amino group. In the next level, the chlorine as a good leaving group leave the molecule and the final structure obtained.

### Similarity search

**Fig. 3:**
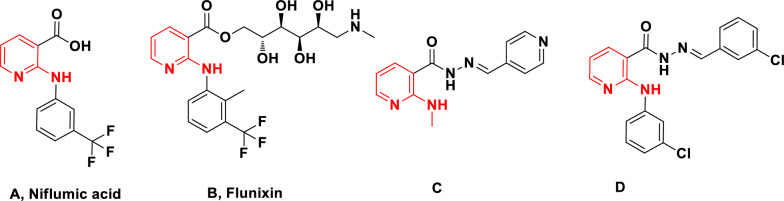
Chemical structures of COX inhibitors with pyridine structure

VS has been routinely employed in modern drug discovery companies, which represents a fast and cost-effective approach to identifying novel biological targets or hit structures. The similarity search is a reliable ligand-based method that determines the similarity of targeted derivatives with biologically active agents. In the current study, similarity search analyses were applied to several libraries, and the results showed that the 2-anilino nicotinic acids can be ideal COX-1 and COX-2 inhibitors. Pyridine core is present in niflumic acid (A) and flunixin (B) as traditional NSAIDs belonging to the class of fenamates. Niflumic acid can inhibit both PLA2 as well as COX-2, thereby acting as a nonsteroidal anti-inflammatory and pain reduction agent [30]. Compounds C and D (Fig. 3) were also synthesized regarding the parental structure of niflumic acid. Compound C bearing 4-pyridyl moiety attached to the imine functional group of nicotinic acid reduced the inflammation to 80% at 1-h postdrug administration [31]. Compound D with lipophilic chloro substituent at *meta* position improved the anti-inflammatory activities with a 95.37 ± 4.45% reduction in inflammation after 30 min of administration [32]. Also, metal complexes of nonsteroidal anti-inflammatory agents bearing niflumic acid were introduced [33–35]. Considering the above results and the similarity in these structures, synthesized derivatives were proposed as possible COX inhibitors. Some evidence suggests that the nicotinic acid group possesses a pharmacophoric character to inhibit COX.

### Docking studies

Regarding the similarity of previously reported COX inhibitors with the designed structure, molecular docking assessments using the Schrodinger package using induced fit docking [36] were executed to study the binding mode of all derivatives within the COX-1 and COX-2 active sites. First, the docking validation was performed against COX-1 (3KK6 in complex with celecoxib) and COX-2 (5KIR in complex with rofecoxib), with the native crystallography ligands. The lowest energy pose related to each enzyme was compared with the coordination of crystallography ligands and recorded the RMSD value less than 2 Å. Next, the same docking procedures were applied for all derivatives, and the results of binding energies and detailed interactions were summarized in Tables 1 and 2.

**Table 1:** Details interactions of synthesized compounds against COX1

Ligand	GlideScore (kcal/mol)	Moiety of ligand	Type of interaction	Residue
1	− 7.500	COOH	H-bound	Arg120
		COOH	H-bound	Arg120
		COOH	H-bound	Tyr355
		Phenyl	Pistacking	Tyr355
2	− 7.811	COOH	H-bound	Arg120
		COOH	H-bound	Arg120
		COOH	H-bound	Tyr355
		Pyridine	H-bound	Ser353
3	− 7.542	COOH	H-bound	Arg120
4	− 7.009	COOH	H-bound	Arg120
		COOH	H-bound	Tyr355
		Phenyl	p stacking	Tyr355
5	− 8.096	COOH	H-bound	Arg120
		COOH	H-bound	Tyr355
6	− 7.525	COOH	H-bound	Arg120
		COOH	H-bound	Tyr355
7	− 5.755	COOH	H-bound	Arg120
		COOH	H-bound	Arg120
		Phenyl	stacking	Tyr355
8	− 8.152	COOH	H-bound	Ser530
9	− 7.912	COOH	H-bound	Arg120
		COOH	H-bound	Tyr355
		2-Cl	Halogen bond	His90
10	− 8.892	COOH	H-bound	Arg120
		COOH	H-bound	Tyr355
11	− 7.304	COOH	H-bound	Arg120
		COOH	H-bound	Tyr355
		Phenyl	stacking	Tyr355
		4-Cl	Halogen bond	Ile517
		4-Cl	Halogen bond	Phe518
12	− 7.903	COOH	H-bound	Arg120
		COOH	H-bound	Arg120
		COOH	H-bound	Tyr355
		Pyridine	H-bound	Ser353
		Pyridine	-cation	Tyr
		4-Cl	Halogen bond	His90
13	− 7.941	COOH	H-bound	Arg120
		COOH	H-bound	Arg120
		COOH	H-bound	Tyr355
		4-Cl	Halogen bond	Phe518
14	− 7.903	COOH	H-bound	Tyr385
15	− 7.002	COOH	H-bound	Arg120
		COOH	H-bound	Arg120
		Phenyl	stacking	Tyr355
		4-Br	Halogen bond	Phe518
16	− 7.162	COOH	H-bound	Arg120
		COOH	H-bound	Arg120
		COOH	H-bound	Tyr355
		Phenyl	stacking	Tyr355
17	− 7.377	COOH	H-bound	Arg120
		COOH	H-bound	Arg120
		Phenyl	stacking	Tyr355
18	− 7.857	COOH	H-bound	Arg120
		COOH	H-bound	Tyr355
19	− 7.051	COOH	H-bound	Arg120
		COOH	H-bound	Arg120
		COOH	H-bound	Tyr355
		NO_2_	H-bound	Ile517
		NO_2_	H-bound	Phe518
		Phenyl	stacking	Tyr355
20	− 5.944	COOH	H-bound	Arg120
		COOH	H-bound	Tyr385
21	− 8.617	COOH	H-bound	Ser530 Tyr385 Tyr355
		COOH	H-bound	
		naphtyl	stacked	
22	− 7.932	COOH	H-bound	Arg120 Ar120
		COOH	H-bound	
23	− 8.625	COOH	H-bound	Arg120 Tyr355
		COOH	H-bound

**Table 2:** Details interactions of synthesized compounds against COX-2

Ligand	Docking score (kcal/mol)	Moiety of ligand	Type of interaction	Residue
1	− 7.535	COOH	H-bound	Arg120
		COOH	H-bound	Tyr355
		COOH	H-bound	Arg513
2	− 5.181	COOH	H-bound	Arg120
		COOH	H-bound	Tyr355
3	− 5.573	Pyridine	stacked	Tyr355
		COOH	H-bound	Arg513
		Phenyl	stacked	Trp387
4	− 7.787	COOH	H-bound	Tyr355
5	− 5.873	COOH	H-bound	Arg120
		COOH	H-bound	Tyr355
6	8.286	COOH	H-bound	Ser530
		Pyridine	stacked	Trp387
7	− 7.668	–	–	–
8	− 8.568	Pyridine	stacked	Phe518
		Phenyl	stacked	His90
9	− 7.698	COOH	H-bound	Tyr355
10	− 8.785	3-Cl	Halogen bound	Ser530
		3-Cl-phenyl	stacked	Trp387
		Pyridine	stacked	Tyr355
		COOH	H-bound	Arg513
11	− 7.803	4-Cl	Halogen bound	Ser530
		4-Cl	Halogen bound	Tyr385
12	− 8.247	COOH	H-bound	Arg120
		COOH	H-bound	Tyr355
		COOH	H-bound	Arg513
		3-Cl	Halogen bound	Ser530
13	− 8.321	Phenyl	stacked	Tyr385
		Phenyl	stacked	Trp384
		Pyridine	H-bound	Ser530
14	− 8.218	COOH	H-bound	Tyr355
		COOH	H-bound	Arg513
		4-Cl	Halogen bound	Ser530
15	− 7.890	COOH	H-bound	Tyr355
		COOH	H-bound	Arg513
		4-Br	Halogen bound	Ser530
16	− 7.613	Meo	Halogen bound	Ser530
17	− 7.793	Meo	Halogen bound	Ser530
		COOH	H-bound	Tyr355
18	− 8.047	COOH	H-bound	Arg120
		COOH	H-bound	Tyr355
		COOH	H-bound	Arg513
		NO_2_	H-bound	Ser530
19	− 8.285	COOH	H-bound	Tyr355
		NO_2_	H-bound	Ser530
20	− 7.995	COOH	H-bound	Arg120
		OH	H-bound	Ser530
21	− 7.380	Pyridine	H-bound	Tyr355
		COOH	H-bound	Arg513
22	− 6.333	COOH	H-bound	Arg513
		COOH	H-bound	Arg513
23	− 8.685	Phenyl	-cation	Arg120
		COOH	H-bound	Ser530

The molecular docking studies of 1–23 against COX-1 showed the binding energy range from − 5.94 to − 8.62 kcal/mol (Table 1). Assessments of all designed molecules exhibited that COOH moiety participated in H-bound interactions with residues of COX-1 active sites such as Arg120, and/or Tyr355 and/or Tyr385. This strong interaction provided preliminary potency against COX-1. In these derivatives, the most potent inhibitor against COX-1 were 10 (GlideScore = − 8.892), 23 (GlideScore = − 8.625), and 21 (GlideScore = − 8.617).

The docking results of all derivatives against COX-2 are exhibited in Table 2. As can be seen, synthesized derivatives recorded binding energy of -8.785 to -5.181 kcal/mol against COX-2. In most cases, the COOH group of these derivatives participated in H-bound interaction with the binding pocket of the enzyme. The active inhibitors were 10 (Docking score =—8.785) > 23 (Docking score = − 8.685**)** > 19 (Docking score = − 8.285).

Overall, it can be seen that compound **2** bearing 2-methyl phenyl followed by derivatives **3** containing *3*-methyl phenyl and **5** (2,3-di methyl phenyl) were the most selective COX-1 inhibitors, while derivative **7** (2,4-dimethyl phenyl) were the most selective COX-2 inhibitor.

### Molecular dynamics simulations

**Fig. 4:**
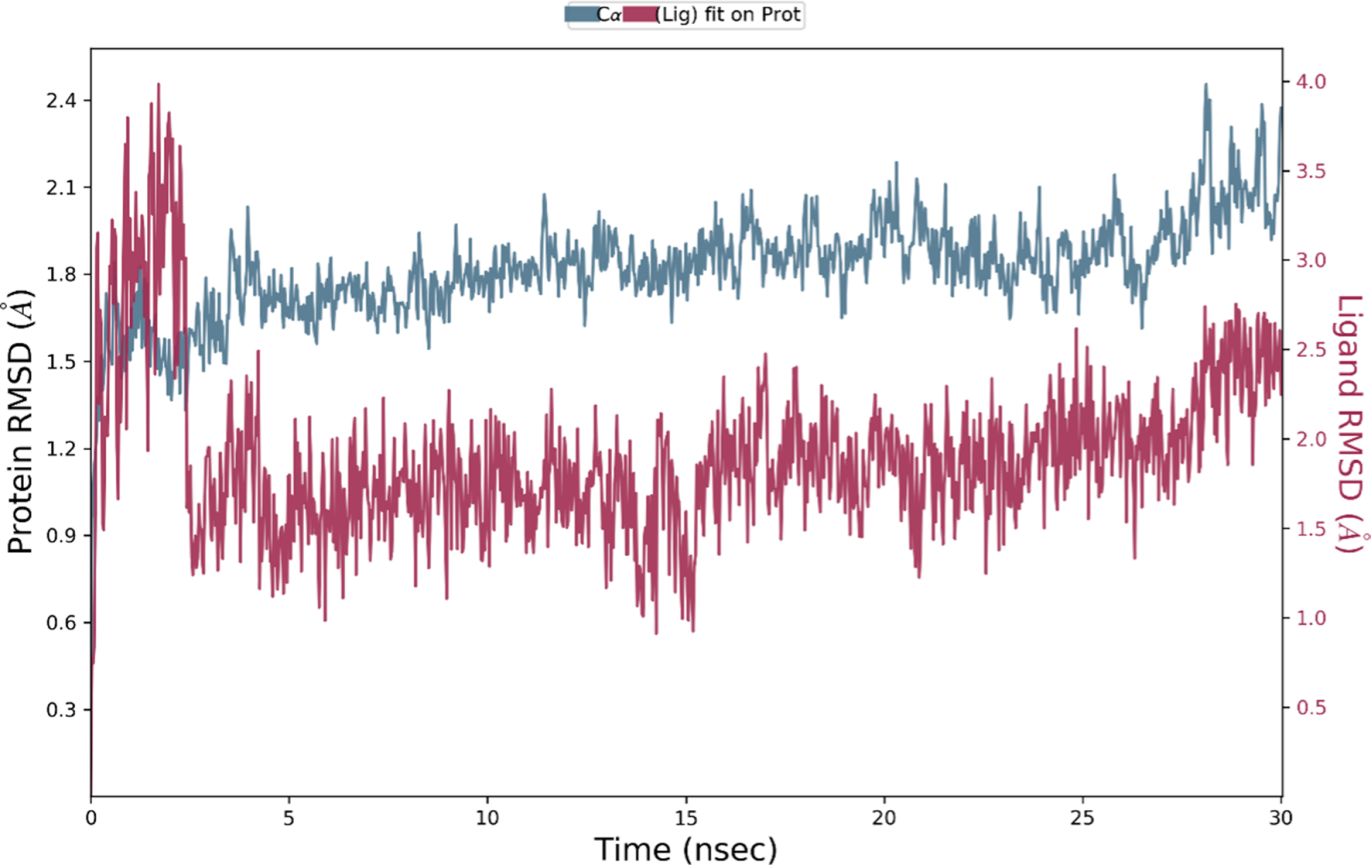
RMSD plot of the COX-1 in complexed compound 10 in the MD simulations time. RMSD values of the Ca atoms of the protein are depicted in blue, and ligand-complex values are exhibited in red

The major weakness of the docking algorithms is the lack or little consideration of the flexibility of the protein during the binding operation between the ligand and the protein [37]. This does not allow an optimal co-adaptation of the ligand and the receptor. MD simulations can handle the flexibility of the protein. Regarding derivative 10 is the most potent COX-1 and COX-2 inhibitor, MD simulations of this derivative against two enzymes were examined. The RMSD results of MD simulation studies against COX-1 at 30 ns are exhibited in Fig. 4. As can be seen, the complex reached stability over 5 ns with slight fluctuations afterward, which is a good indication of system stability with the value of 1.2 Å.

**Fig. 5:**
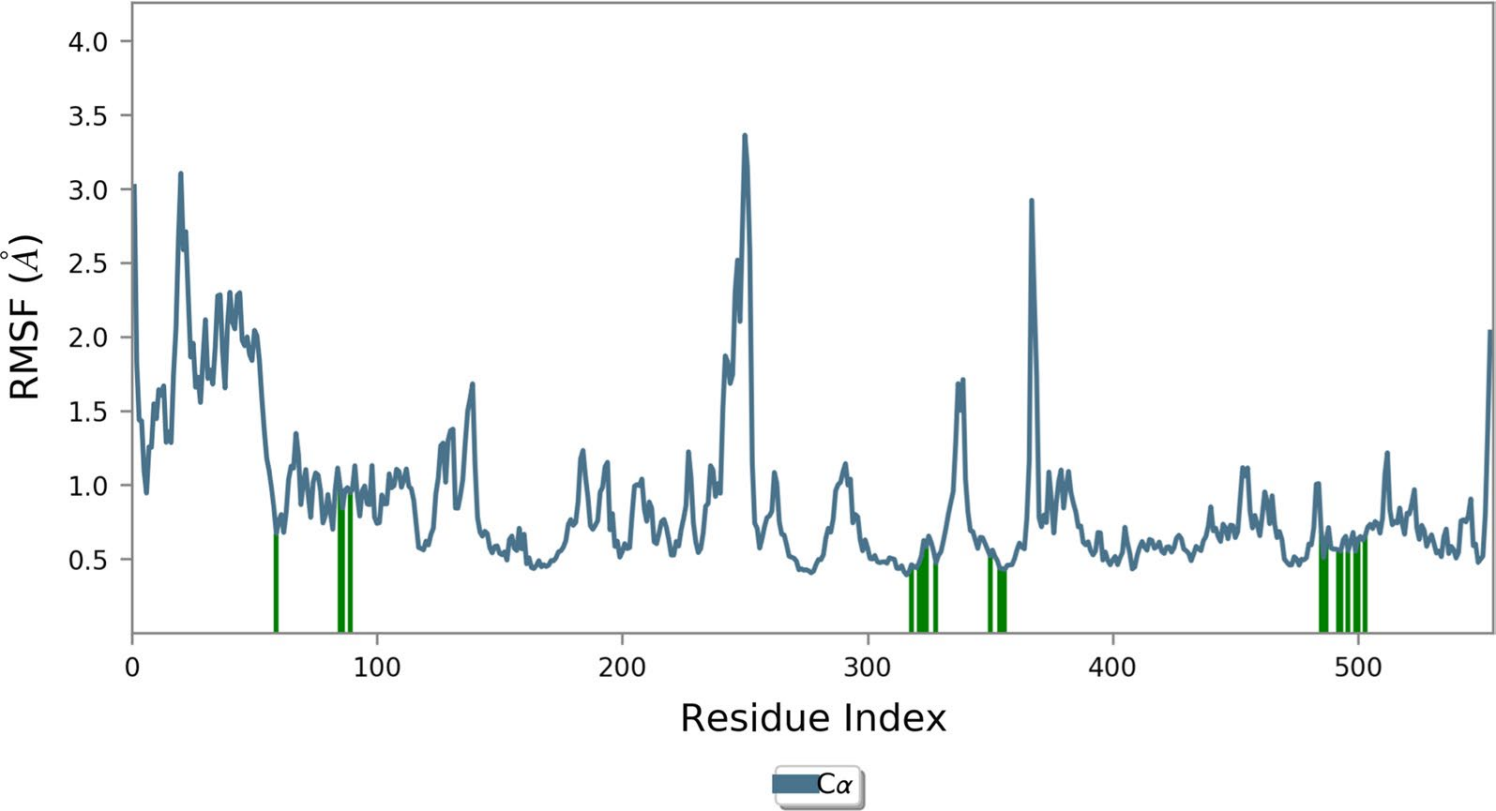
The RMSF graph of COX-1 in complex with 10

Root mean square fluctuation (RMSF) studies are suitable for determining the protein's average mobility within the MD simulations. RMSF of the complex was analyzed to explore the flexibility of individual residues. As can be seen in Fig. 5, the conformational changes of the amino acids in the active site are very small, confirming the formation of stable interactions with the protein during MD simulation.

The MD trajectories were also analyzed to calculate the number and types of interactions formed during the MD simulation. Additional file 1: Fig. S2 shows the hydrogen-bound interactions between Arg120 and COOH moiety of derivative 10. This moiety exhibited another H-bound interaction with Tyr355 for 82% of MD duration. Also, the H-bound interaction between the pyridine ring and Ser530 was seen in around 50% of cases. It was also evident from Additional file 1: Fig. S3 that the number of hydrogen bonds was steadily maintained throughout the simulation for protein with selected compounds.

**Fig. 6:**
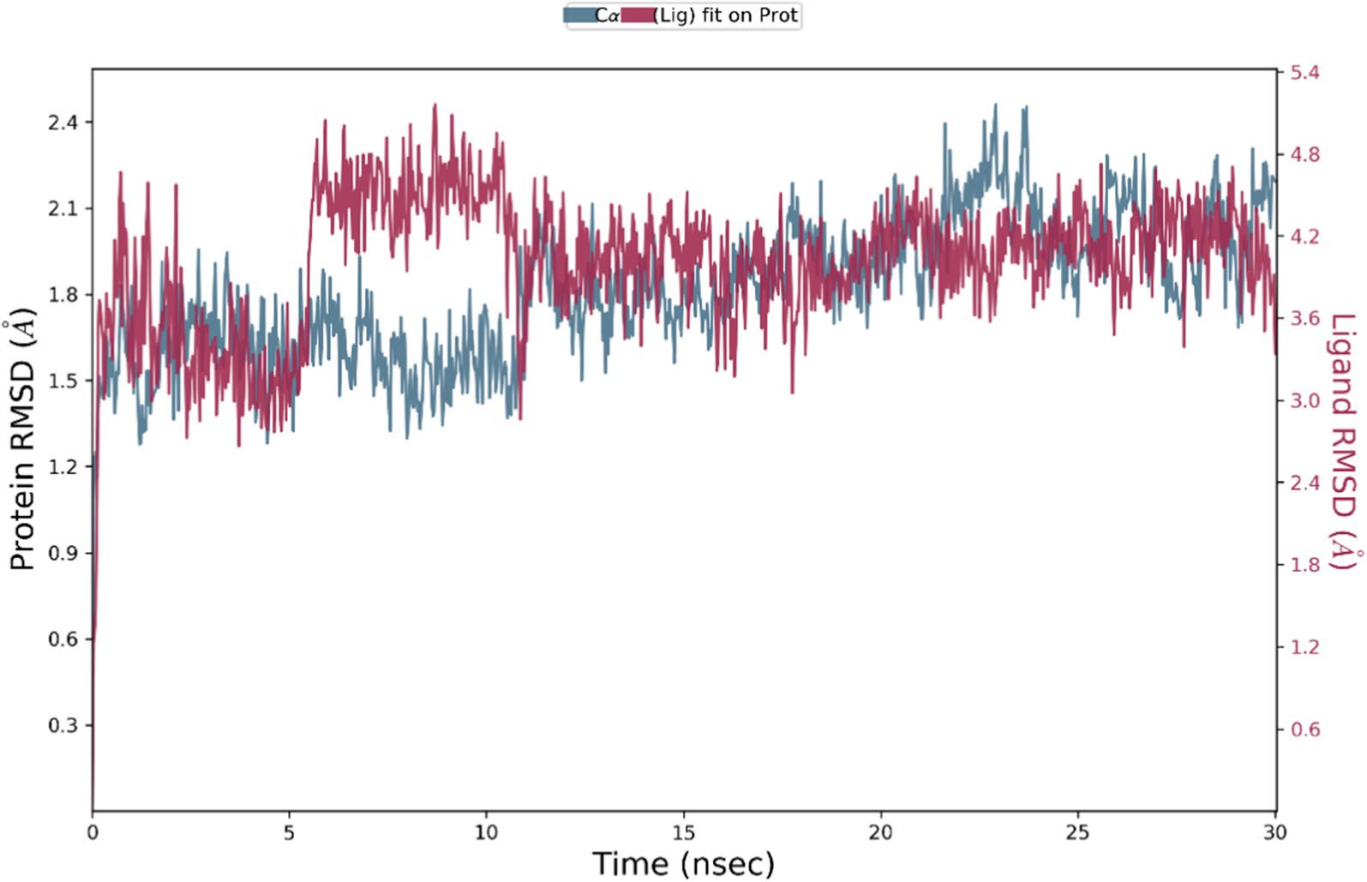
RMSD plot of the COX-2 in complexed compound 10 in the MD simulation time. RMSD values of the Ca atoms of the protein are depicted in blue, and ligand-complex values are exhibited in red

The RMSD of the COX-2 with compound 10 backbone from its initial to final conformation was applied over 30 ns MD simulation to study the stability of the protein–ligand complex. The RMSD simulation showed COX-2 complexed with ligand got overall stability after 11 ns of MD simulations time with RMSD stabilizing at an average of 1.80 Å (Fig. 6). So, the RMSD value of the COX-2 complex indicated that the employed simulation time was adequate to obtain an equilibrium structure and was proper to investigate the structural specificity of the ligand–protein complexes.

**Fig. 7:**
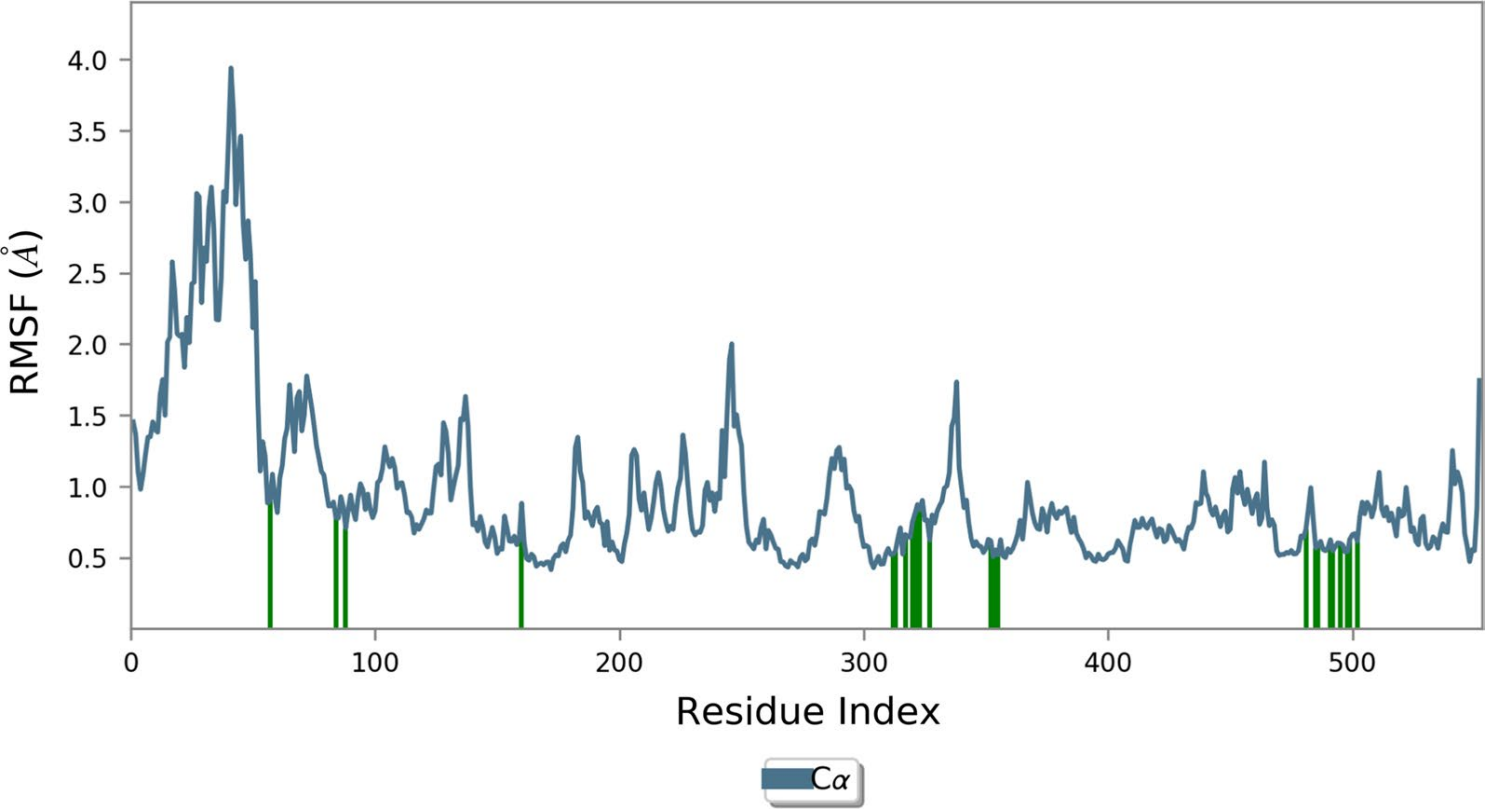
RMSF plot of the COX-2 residue in complexed with compound 10

The RMSF is useful for characterizing local changes along the protein chain and the flexibility of the protein (Fig. 7). The residue 40–70 indicated areas of the protein that fluctuated the most during the simulation and the residue of the active site did not show significant fluctuations.

In more inspection, Additional file 1: Fig. S3 shows the molecular interactions of COX-2 and compound 10 over the binding site of COX-2 during the whole simulation time. Additional file 1: Fig. S3-a shows compound 10 formed stable three H-bound interactions with Arg120 and Ser530 for most of the simulation time. Interactions that occur more than 30.0% of the simulation time in the selected trajectory (0.00 ns through 30.00 ns) are shown in Additional file 1: Fig. S3. Arg120 participated in important two hydrogen bond interactions with COOH. Also, Ser530 demonstrated H-bound interaction (84%) with pyridine (Additional file 1: Fig. S3).

Overall the multiple hydrogen bonding interactions were presented in the simulation trajectory in the active site of COX-1 and COX-2. Both complexes were stable during the MD simulation time (confirmed with RMSD) with limited fluctuation of the residues of the binding site, ensuring the proper fitting of compound 10 in the COXs active sites.

## Conclusion

In summary, we developed a direct and inexpensive method for the synthesis of 2-anilino nicotinic acids. High yields, short reaction times, easy work-up, absence of any volatile and hazardous organic solvents, and green reaction conditions are some advantages of this protocol. Furthermore, the reaction displayed good functional group tolerance, and product isolation is very straightforward without using non-green and harmful solvents. The in silico assessment was carried out on synthesized compounds and indicated that these derivatives are potent inhibitors of COX-1 and COX-2. Induced fit docking investigation confirmed the important role of COOH and pyridine functional groups in participating in H-bound interactions with the binding site of these enzymes. It was shown that derivative **10** bearing *meta* chloro moiety was the most potent COX-1 and COX-2 inhibitors. MD study disclosed that compound **10** was stabilized in the binding pocket of COX-1 and COX-2 and exhibited pronounced interactions with essential residues of enzymes through the H-bound interactions with important residues at the active site of enzymes. These results help to design novel potent COX inhibitors.

## Experimental

### Instruments and characterization

All chemicals were purchased from Merck, Fluka and Sigma-Aldrich companies and were used without further purification, except for benzaldehyde, which was used as a fresh distilled sample. Analytical thin layer chromatography (TLC) for monitoring reactions was performed using Merck 0.2 mm silica gel 60 F-254 Al-plates using ethyl acetate and n-hexane as eluents. Melting points were determined in open capillaries using an Electrothermal 9100 instrument. Infrared (IR) spectra were acquired on a Shimadzu FT-IR-8400S spectrometer. ^1^H NMR (500 MHz) and ^13^C NMR (125 MHz) spectra were recorded on a Bruker DRX-500 Avance spectrometers with CDCl_3_ as solvent at ambient temperature and tetramethylsilane (TMS) as the internal standard. All chemical shifts are given relative to TMS. All yields refer to the isolated products.

### General procedure for the synthesis of 2-Anilino Nicotinic Acids

A mixture of Aromatic aniline (1.5mol), 2-chloronicotinic acid (1mol) was heated in an oil bath with stirring at 120 °C for an appropriate time (see Additional file 1: Table S1). After the completion of the reaction, as monitored by thin-layer chromatography (TLC), the reaction mixture was subsequently cooled to room temperature, and cold water was added to the reaction mixture. Then the precipitated solid was collected by filtration and dried. If necessary, the products can be purified by recrystallization from ethanol. All of the products were known compounds. The spectral data for compound 3y were provided in Additional file 1: Figs. S4–S8.

### Similarity-based analog searching

To find an ideal biological target for this set of compounds, ligand-based similarity search on several libraries was accomplished, including PubChem similarity search (https://pubchem.ncbi.nlm.nih.gov/) SwissTargetPrediction (http://www.swisstargetprediction.ch/), SEA Search Server (https://sea.bkslab.org/), MolTarPred (https://moltarpred.marseille.inserm.fr/). These databases applied different searching approaches, such as fingerprint and shape-based similarity pharmacophores to find the most similar bioactive agents compared to the synthesized compounds.

### Molecular docking

The molecular docking investigation of all derivatives was performed using the maestro molecular modeling platform (Schrödinger 2018‐4 suite). X-ray crystallographic structures of COX-1 (PDB ID: 3KK6) and COX-2 (PDB ID: 5KIR) were downloaded from the PDB website (https://www.rcsb.org/). For each enzyme protein preparation wizard was used to remove water molecules and co-crystallized atoms from the protein and prepare the receptor. Moreover, heteroatom states were generated at pH: 7.4 by EPIK, and H-bonds were assigned using PROPKA at the same pH. 2D structure of ligands was drawn in Hyperchem, energy minimized using, molecular mechanics and molecular quantum approaches. Next, the ligand preparation wizard was used to prepare the ligand using the OPLS_2005 force field. All compounds were docked into the binding sites using induced fit docking tasked to report the best pose per ligand with flexible ligand sampling and extra precision.

### Molecular dynamic simulation

The molecular simulation was performed using the Desmond v5.3 (Schrödinger 2018‐4 suite). To build the system for MD simulation, the protein–ligand complexes were solvated with SPC explicit water molecules and placed in the center of an orthorhombic box of appropriate size in the periodic boundary condition. Sufficient counter‐ions and a 0.15 M solution of NaCl were also utilized to neutralize the system and to simulate the real cellular ionic concentrations, respectively. The MD protocol involved minimization, pre-production, and finally, production MD simulation steps. In the minimization procedure, the entire system was allowed to relax for 2500 steps by the steepest descent approach. Then the temperature of the system was raised from 0 to 300 K with a small force constant on the enzyme in order to restrict any drastic changes. MD simulations were performed via NPT (constant number of atoms, constant pressure i.e. 1.01325 bar, and constant temperature i.e. 300 K) ensemble. The Nose‐Hoover chain method was used as the default thermostat with 1.0 ps interval and Martyna‐Tobias‐Klein as the default barostat with 2.0 ps interval by applying an isotropic coupling style. Long‐range electrostatic forces were calculated based on the particle‐mesh‐based Ewald approach with the cut‐off radius for Columbia forces set to 9.0 Å. Finally, the system was subjected to produce MD simulations for 100 ns for each protein–ligand complex. During the simulation, every 30 ns of the actual frame was stored. The dynamic behavior and structural changes of the systems were analyzed by the calculation of the RMSD and RMSF.

### Supplementary Information

**Additional file 1:** **Figure S1.** Proposed mechanism for the formation of aryl amino nicotinic acids. **Figure S2.** **a** 2D representation of ligand-residue interactions that occur at least 30% of simulation time at the equilibrated phase of MD simulation b) Timeline rendering of numbers of hydrogen bonds formed COX-1 and derivative 10. **Figure S3.** 2D representation of ligand-residue interactions that occur at least 30% of simulation time at the equilibrated phase of MD simulation, including COX-2 with compound 10 (**a**). Timeline rendering of interacting residues during the whole simulation time in COX-2 complexed with compound 10 (**b**). **Figure S4.** FTIR spectrum of (3y). **Figure S5.** ^1^HNMR spectrum of (3y) in DMSO-d_6_. **Figure S6**. ^1^HNMR spectrum of (3y) in DMSO-d_6_ (Expanded aromatic region). **Figure S7**. ^13^C-NMR spectrum of (3y) in CDCl_3_ & DMSO-d_6_. **Figure S8.** GC/Ms spectrum of (3y). **Table S1.** Optimization conditions for the synthesis of (3a)^a^. **Table S2.** Four-component synthesis of different 2-arylaminonicotinic acids (3a–y) via condensation of various aromatic amines (2a–y), under solvent-free conditions^a^. **Table S3**. Comparison of results for the synthesis of 3a with other methods.

## Data Availability

The authors confirm that the data supporting the findings of this study are available within the article and its supplementary materials. All relevant data are within the paper and its Supporting Information files.
