# Supplementary material for: Environmentally friendly catalyst- and solvent-free synthesis of 2-anilino nicotinic acids derivatives as potential lead COX inhibitors
Source: BMC Chem. 2023 Nov 20;17(1):160. doi: 10.1186/s13065-023-01078-y (PMC10662667; doi:10.1186/s13065-023-01078-y)
Supplement: Supplementary file 1 — Additional file 1: Figure S1. Proposed mechanism for the formation of aryl amino nicotinic acids. Figure S2. a 2D representation of ligand-residue interactions that occur at least 30% of simulation time at the equilibrated phase of MD simulation b) Timeline rendering of numbers of hydrogen bonds formed COX-1 and derivative 10. Figure S3. 2D representation of ligand-residue interactions that occur at least 30% of simulation time at the equilibrated phase of MD simulation, including COX-2 with compound 10 (a). Timeline rendering of interacting residues during the whole simulation time in COX-2 complexed with compound 10 (b). Figure S4. FTIR spectrum of (3y). Figure S5. 1HNMR spectrum of (3y) in DMSO-d6. Figure S6. 1HNMR spectrum of (3y) in DMSO-d6 (Expanded aromatic region). Figure S7. 13C-NMR spectrum of (3y) in CDCl3 & DMSO-d6. Figure S8. GC/Ms spectrum of (3y). Table S1. Optimization conditions for the synthesis of (3a)a. Table S2. Four-component synthesis of different 2-arylaminonicotinic acids (3a–y) via condensation of various aromatic amines (2a–y), under solvent-free conditionsa. Table S3. Comparison of results for the synthesis of 3a with other methods. [file 13065_2023_1078_MOESM1_ESM.docx]

**Environmentally Friendly Catalyst- and** **Solvent-Free Synthesis of 2-Anilino Nicotinic Acids Derivatives as Potential Lead COX Inhibitors**

Mahsa Yarhorhosseini^a^, Shahrzad Javanshir^*a^, Ahmad Shahir Sadr^*b^, Milad Noori^a^, Navid Dastyafteh^a^, Maryam Esmkhani^a^ Aida Iraji^*c,d^, Mohammad Mahdavi^e^,

*^a^ Heterocyclic chemistry Research Laboratory, Department of Chemistry, Iran University of Science and Technology, Tehran 16846-13114, Iran. E-mail:* [*shjavan@iust.ac.ir*](mailto:shjavan@iust.ac.ir)

*^b^Bioinformatics Research Center,‎Cheragh Medical institute& Hospital, Kabul, Afghanistan*

*^c^Stem Cells Technology Research Center, Shiraz University of Medical Sciences, Shiraz, Iran*

*^d^ Central Research Laboratory, Shiraz University of Medical Sciences, Shiraz, Iran*

*^e^ Endocrinology and Metabolism Research Center, Endocrinology and Metabolism Clinical Sciences Institute, Tehran University of Medical Sciences, Tehran, Iran*

Additional file 1: Figure S1. Proposed mechanism for the formation of aryl amino nicotinic acids


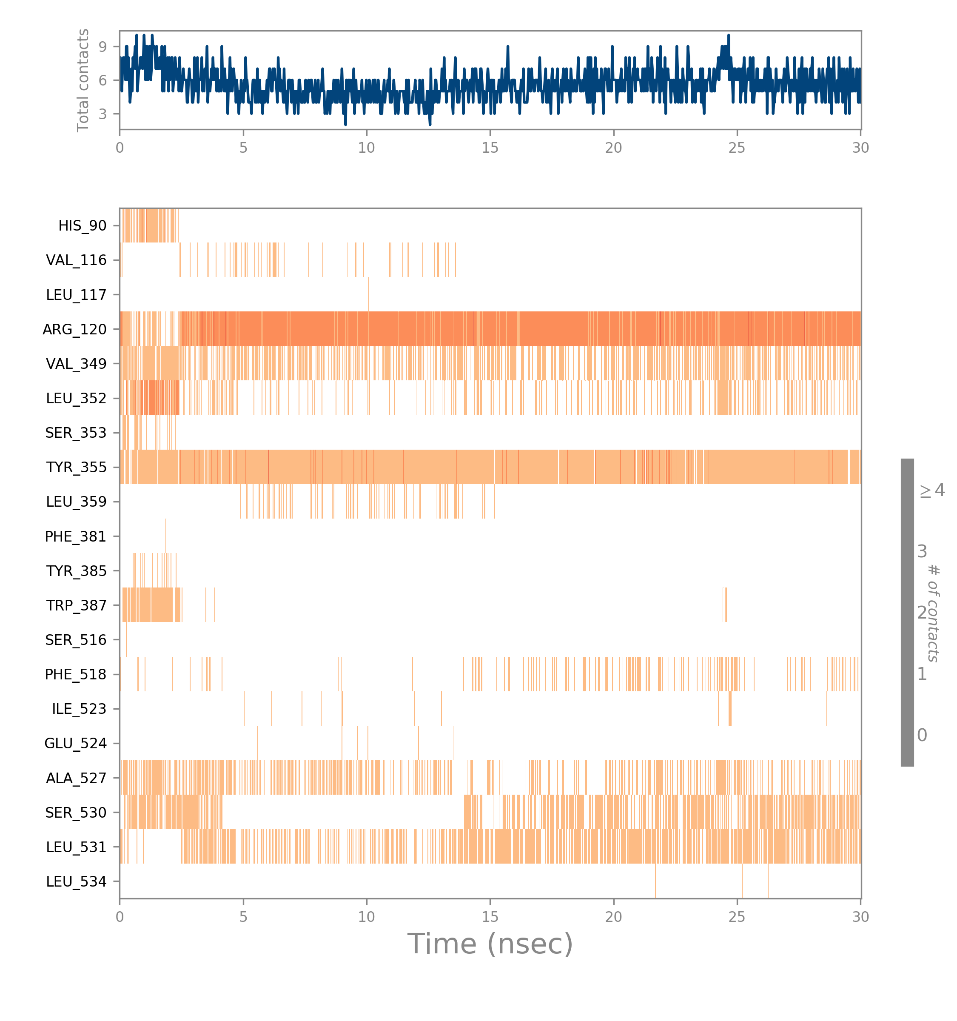

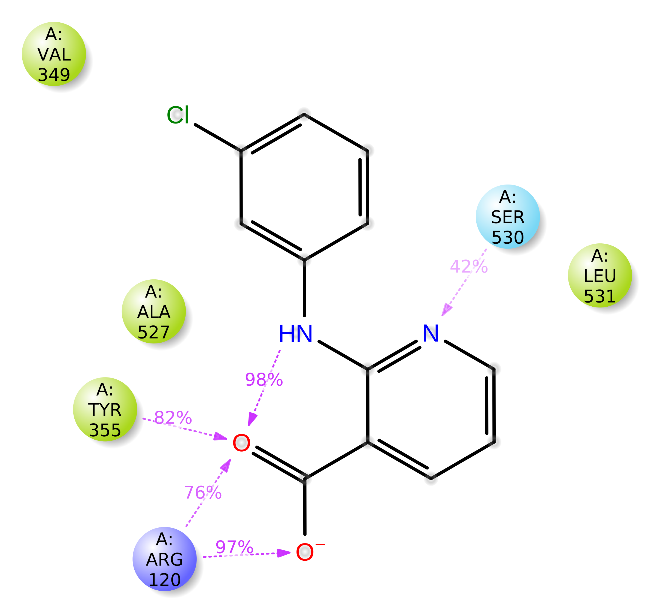


**a)**

**b)**

Additional file 1: Figure S2. a) 2D representation of ligand-residue interactions that occur at least 30% of simulation time at the equilibrated phase of MD simulation b) Timeline rendering of numbers of hydrogen bonds formed COX-1 and derivative 10.


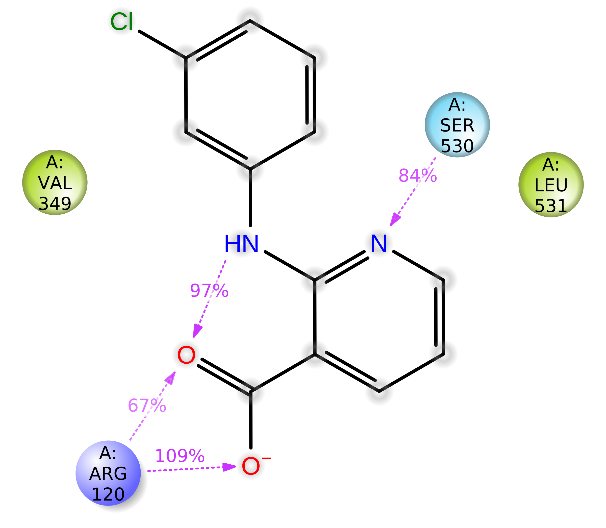

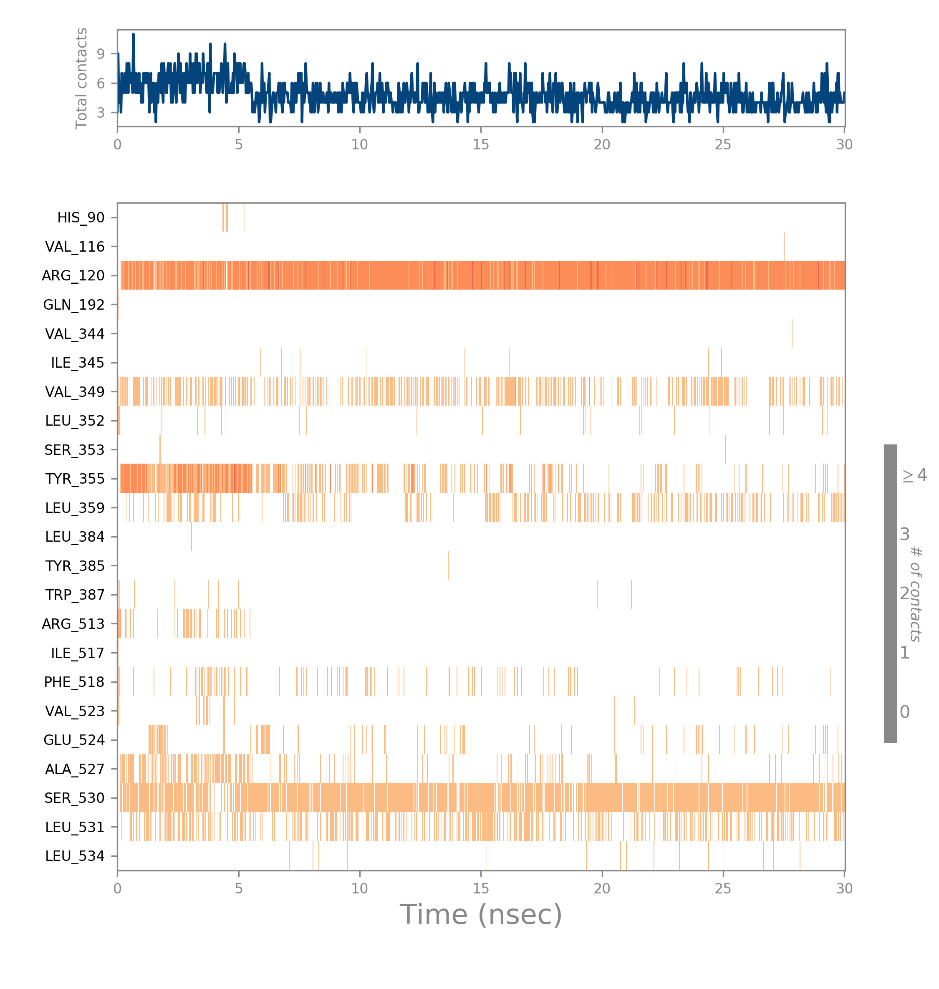


**a)**

**b)**

Additional file 1: Figure S3. 2D representation of ligand-residue interactions that occur at least 30% of simulation time at the equilibrated phase of MD simulation, including COX-2 with compound **10** **(a)**. Timeline rendering of interacting residues during the whole simulation time in COX-2 complexed with compound **10** **(b)**.

**Figure S 2.**

Additional file 1: Table S1. Optimization conditions for the synthesis of (3a)^a^.

| Entry | Ratio of 1/2a | catalyst | Catalyst loading  (mol %) | Solvent | Temp  ( °C ) | Time  (min) | Yield^d^  ( % ) |
| --- | --- | --- | --- | --- | --- | --- | --- |
| 1 | 1:1.5 | Na_2_CO_3_ | 8 | - | 120 | 15 | 60 |
| 2 | 1:1.5 | NEt_3_ | 8 | - | 120 | 15 | 50 |
| 3 | 1:1.5 | CuSO^4^ | 8 | - | 120 | 15 | 75 |
| 4 | 1:1.5 | PTSA^b^ | 8 | - | 120 | 15 | 88 |
| **5** | **1:1.5** | **-** | **-** | **-** | **120** | **15** | **90** |
| 6 | 1:1.5 | - | - | H_2_O | Reflux | 720 | 60 |
| 7 | 1:1.5 | - | - | EtOH | Reflux | 720 | 50 |
| 8 | 1:1.5 | - | - | n-Hexanol | Reflux | 720 | 67 |
| 9 | 1:1.5 | - | - | PEG-400^c^ | Reflux | 120 | 50 |
| 10 | 1:1.5 | PTSA | 8 | H_2_O | Reflux | 300 | 60 |
| 11 | 1:1.5 | PTSA | 8 | n-Hexanol | Reflux | 120 | 78 |
| 12 | 1:1.5 | - | - | - | 70 | 30 | 80 |
| 13 | 1:1.5 | - | - | - | 100 | 20 | 82 |
| 14 | 1:2 | - | - | - | 120 | 15 | 85 |
| 15 | 1:1 | - | - | - | 120 | 20 | 60 |

^a^  Reaction conditions: aniline (1/5mol), 2-chloronicotinic acid (1mol)

^b^ p-Toluenesulfonic acid

^c^ Polyethylene glycol

^d^ The yields refer to the isolated product.

Additional file 1: Table S2. Four-component synthesis of different 2-arylaminonicotinic acids (3a-y) via condensation of various aromatic amines (2a-y), under solvent-free conditions. ^a^

| Entry | Aniline | Product | Time (min) | Yield^b^ ( % ) | Mp (°C) |
| --- | --- | --- | --- | --- | --- |
| **1** |   **2a** |   **3a** | 15 | 90 | 150  [149-150]^3^ |
| **2** |   **2b** |   **3b** | 10 | 95 | 164-165  [165]^18^ |
| **3** |   **2c** |   **3c** | 15 | 90 | 157  [158]^3^ |
| **4** |   **2d** |   **3d** | 10 | 97 | 203  [203-204]^18^ |
| **5** |   **2e** | **3e** | 20 | 92 | 237  [236-237]^18^ |
| **6** | **2f** |   **3f** | 30 | 40 | 209-212  [209-211]^18^ |
| **7** |   **2g** |   **3g** | 20 | 95 | 225-227  [225]^19^ |
| **8** |   **2h** | **3h** | 30 | 45 | 256  [257-258]^18^ |
| **9** |   **2i** |   **3i** | 30 | 50 | 213-215  [212-214]^18^ |
| **10** |   **2j** |   **3j** | 20 | 92 | 198-201  [199-201]^20^ |
| **11** |   **2k** |   **3k** | 20 | 90 | 208  [207-208]^18^ |
| **12** |   **2l** |   **3l** | 40 | 42 | 256  [256-258]^21^ |
| **13** |   **2m** |   **3m** | 45 | Trace | 264-267  [266-267]^20^ |
| **14** |   **2o** | **3o** | 30 | 60 | 258-260  [260]^22^ |
| **15** |   **2p** |   **3p** | 15 | 92 | 210-212  [211]^21^ |
| **16** |   **2q** |   **3q** | 20 | 80 | 205-208  [207-208]^20^ |
| **17** |   **2r** |   **3r** | 20 | 85 | 197-198  [195-197]^18^ |
| **18** |   **2s** |   **3s** | 40 | 88 | 242-244  [245]^23^ |
| **19** |   **2t** |   **3t** | 60 | Trace | 274-275  [272-274]^24^ |
| **20** |   **2u** |   **3u** | 25 | 82 | 230-232  [230-232]^20^ |
| **21** |   **2w** |   **3w** | 30 | 72 | 191-194  [193-194]^18^ |
| **22** |   **2x** |   **3x** | 20 | 88 | 178  [175-176]^20^ |
| **23** |   **2y** |   **3y** | 120 | 50 | 230  [227-229]^25^ |

^a^  Reaction conditions: Aromatic aniline (1/5mol), 2-chloronicotinic acid (1mol)

^b^ The yields refer to the isolated product

Additional file 1: Table S3. Comparison of results for the synthesis of 3a with other methods.

| Entry | Ratio of 1/2a | Condition | Temp  ( °C ) | Time  (min) | Yield^a^  ( % ) |
| --- | --- | --- | --- | --- | --- |
| 1 | 1:2 | K_2_CO_3_ as a base, H_2_O | 150 | 120 | 85[^3^] |
| 2 | 1:2 | Microwave irradiation, H_2_O | 120 | 60 | 76 [^18^] |
| 3 | 1:2 | Cu as catalyst, Xylene | Reflux | 3600 | 78 [^7^] |
| 4 | 1:3 | K_2_CO_3_/Cu, DMF | Reflux | 120 | 81 [^26^] |
| **5** | **1:1.5** | **Solvent and catalyst free** | **120** | **15** | **90**  **[This work]** |

^a^ The yields refer to the isolated product

**Chemical characterization of (3y)**

White crystals, mp 223-225°C, isolated yield: %92.

IR (KBr) cm^-1^: 3238, 2300-2775, 2453, 1677, 1579, 1508, 1454, 1319, 1240, 1164, 1122, 1020, and 792. ^1^H NMR (300 MHz, DMSO-*d_6_*) δ (ppm): 2.34 (s, 3H, Me), 6.87 (dd, 1H, *J*= 7.5 and 4.8 Hz, Pyrid-H), 7.34-7.41 (m, 2H, Arom-H), 8.25 (d, 1H, J= 7.8 Hz, Pyrid-H), 8.32 (d, 1H, *J*=4.5, Arom-H), 8.37 (d, 1H, *J*= 7.5 Hz, Pyrid-H), 10.32 (s, 1H, -NH) and 13.51 (brs, 1H, -COOH). ^13^C-NMR (125 MHz CDCl_3_ & DMSO-*d_6_*) δ (ppm): 13.1, 107.4, 113.11, 119.7, 123.5, 125.00, 125.08, 125.7, 127.3 (m, CF_3_), 139.1, 140.0, 151.8, 155.6, 169.0.

C_14_H_11_F_3_N_2_O_2_, MW= 296/24 gr.mol^-1^. M/Z (%): 296(M+), 282, 139, 121, 91.


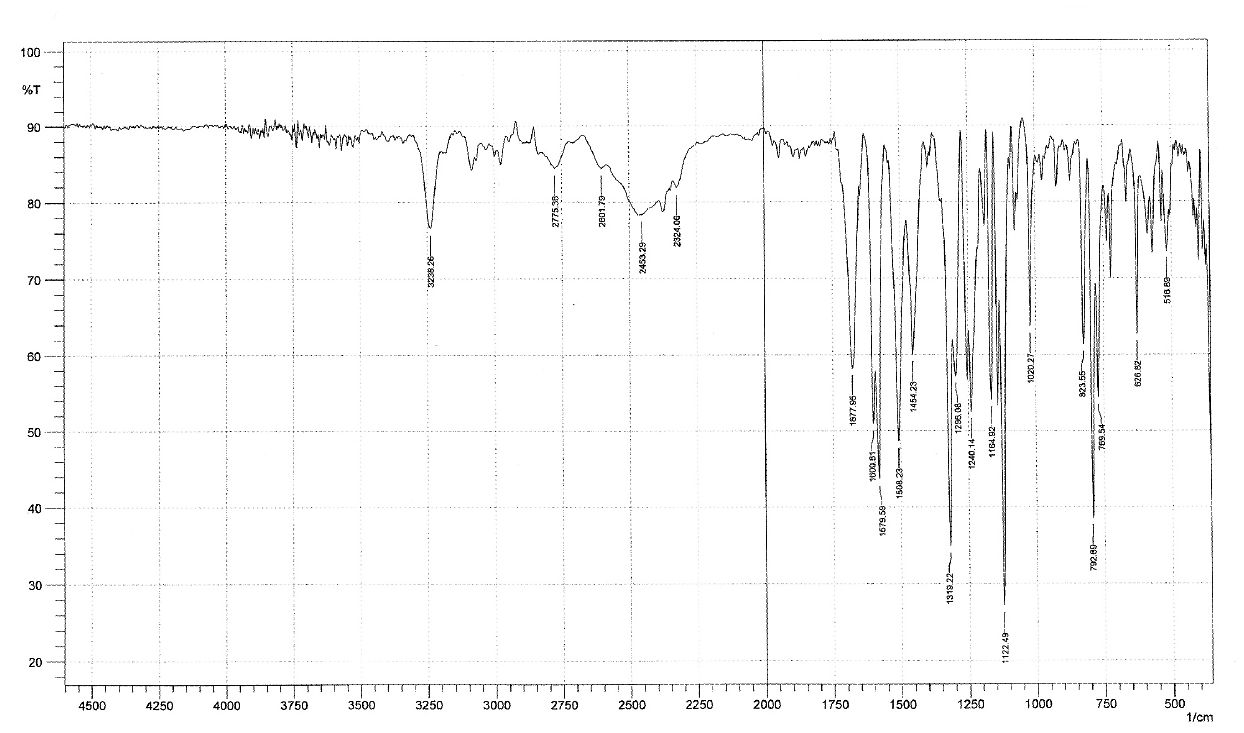

Additional file 1: Figure S4. FTIR spectrum of (3y)


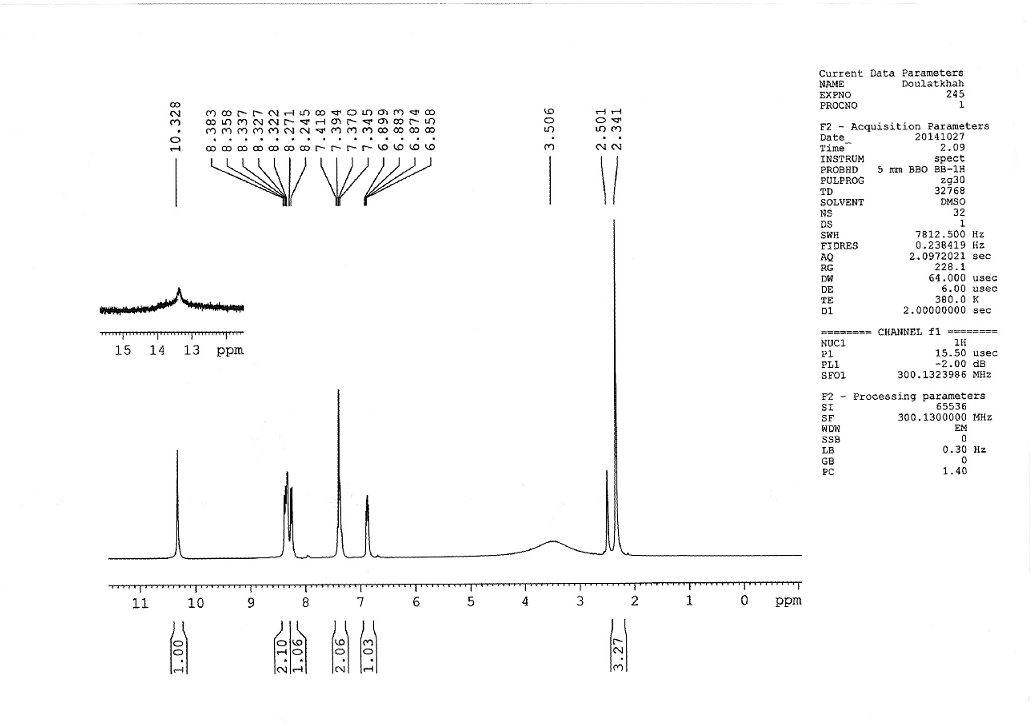

Additional file 1: Figure S5. ^1^HNMR spectrum of (3y) in DMSO-d_6_


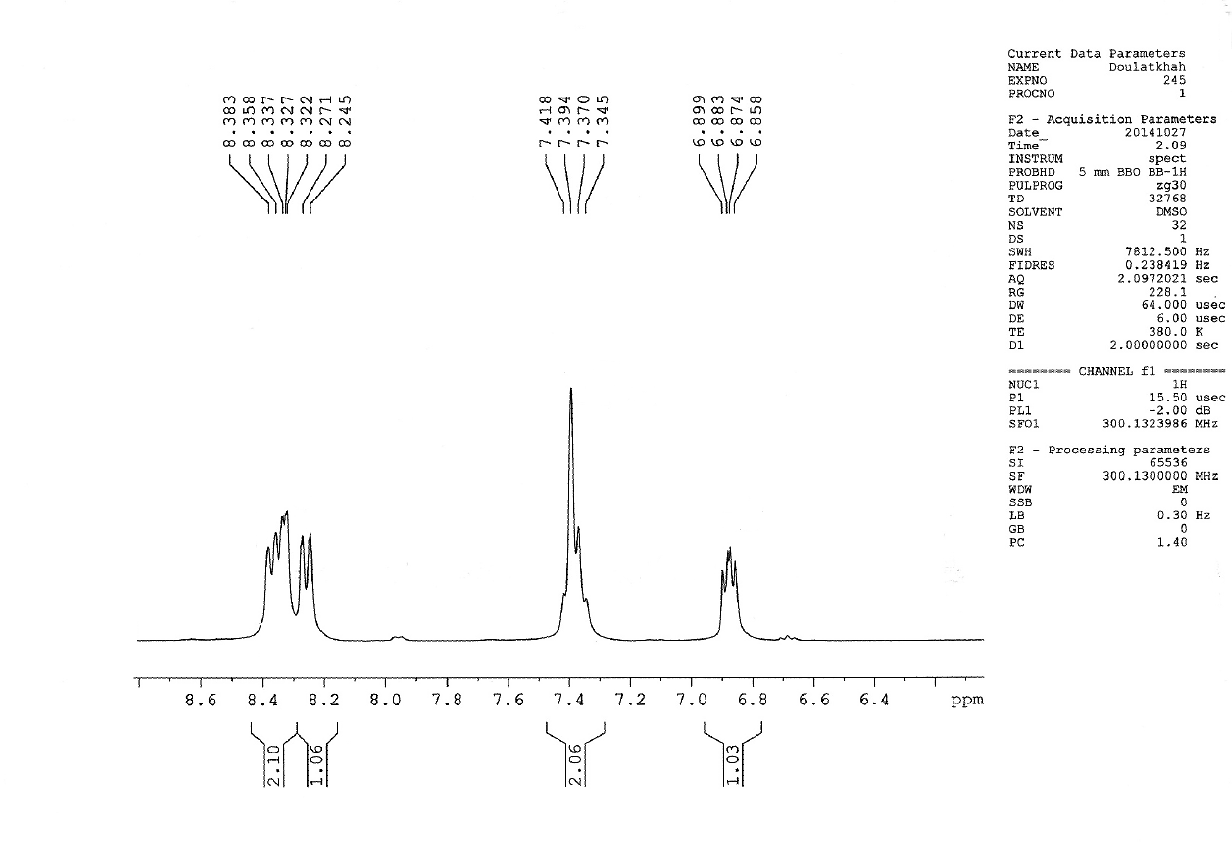

Additional file 1: Figure S6. ^1^HNMR spectrum of (3y) in DMSO-d_6_ (Expanded aromatic region)


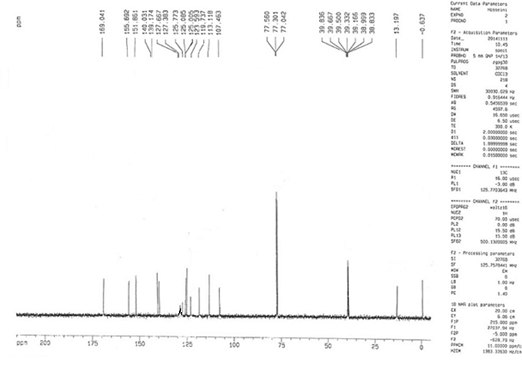

Additional file 1: Figure S7. ^13^C-NMR spectrum of (3y) in CDCl_3_ & DMSO-d_6_


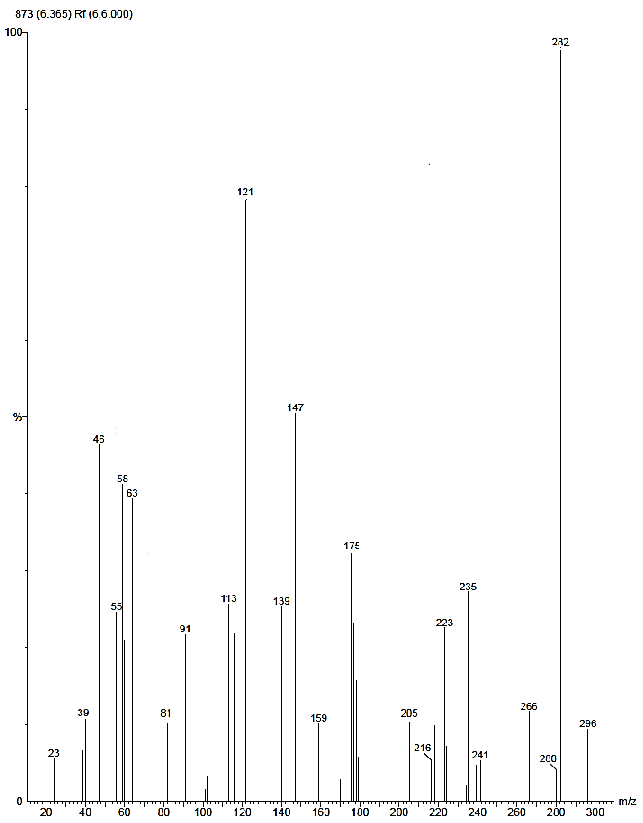


Additional file 1: Figure S8. GC/Ms spectrum of (3y)
